# A Benchmark Protocol for DFT Approaches and Data-Driven Models for Halide-Water Clusters

**DOI:** 10.3390/molecules27051654

**Published:** 2022-03-02

**Authors:** Raúl Rodríguez-Segundo, Daniel J. Arismendi-Arrieta, Rita Prosmiti

**Affiliations:** 1 Institute of Fundamental Physics (IFF-CSIC), Consejo Superior de Investigaciones Científicas, Serrano 123, 28006 Madrid, Spain; raul@iff.csic.es; 2 Atelgraphics S.L., Mota de Cuervo 42, 28043 Madrid, Spain; 3 Department of Chemistry, Ångström Laboratory, Uppsala University, P.O. Box 538, 75121 Uppsala, Sweden; daniel.arismendi@kemi.uu.se

**Keywords:** electronic structure calculations, molecular interactions, benchmark protocol, data-driven modeling, genetic algorithm, ions hydration

## Abstract

Dissolved ions in aqueous media are ubiquitous in many physicochemical processes, with a direct impact on research fields, such as chemistry, climate, biology, and industry. Ions play a crucial role in the structure of the surrounding network of water molecules as they can either weaken or strengthen it. Gaining a thorough understanding of the underlying forces from small clusters to bulk solutions is still challenging, which motivates further investigations. Through a systematic analysis of the interaction energies obtained from high-level electronic structure methodologies, we assessed various dispersion-corrected density functional approaches, as well as ab initio-based data-driven potential models for halide ion–water clusters. We introduced an active learning scheme to automate the generation of optimally weighted datasets, required for the development of efficient bottom-up anion–water models. Using an evolutionary programming procedure, we determined optimized and reference configurations for such polarizable and first-principles-based representation of the potentials, and we analyzed their structural characteristics and energetics in comparison with estimates from DF-MP2 and DFT+D quantum chemistry computations. Moreover, we presented new benchmark datasets, considering both equilibrium and non-equilibrium configurations of higher-order species with an increasing number of water molecules up to 54 for each F, Cl, Br, and I anions, and we proposed a validation protocol to cross-check methods and approaches. In this way, we aim to improve the predictive ability of future molecular computer simulations for determining the ongoing conflicting distribution of different ions in aqueous environments, as well as the transition from nanoscale clusters to macroscopic condensed phases.

## 1. Introduction

The study of water’s properties is one of the most intense research topics [1,2,3,4,5,6,7]. In practice, you can rarely find water in its pure state, and the presence of other molecules is common, which can produce changes in the observed behavior of the water network. This is the case of ions, and depending on their charge and size could be strongly hydrated or disrupt the structure of the surrounding water hydrogen bonding [8,9,10,11,12,13,14,15,16,17,18,19]. Ion hydration is a key point in understanding a variety of physicochemical processes; thus both experimental and theoretical studies are seeking to give a definitive picture of how ions influence the water molecules around them [10,12,14,15,16,18,19,20,21,22,23,24,25,26,27,28,29,30,31,32,33,34,35,36,37]. On the one hand, it has been possible to obtain a clearer approximation of specific structure effects thanks to neutron and X-ray diffraction experiments [4,6,16,38,39]. However, on the other hand, the interpretation of these data strongly depends on the models used to analyze them. Thus, developing potential models of high quality implies the parameterization of rather sophisticated functions of atomic coordinates to properly describe/represent the underlying short- and long-range many-body interactions.

Interaction potentials can be parameterized either from experimental data or first-principles values, with the latter representing a more adequate way to reproduce energies, as they are not biased by the subsequent reinterpretation that must be made of the laboratory data. Indeed, very valuable information has been obtained from numerous quantum chemistry calculations of water and ion–water clusters [30,31,32,33,34,35,36,37,40,41,42,43,44,45,46], at different levels of theory; however, they are usually limited to a few water molecules systems. A systematic analysis of the cluster properties as a function of its size provides a protocol for the selection of the best computational method for modeling ion–water interactions when going from systems in gas to condensed phase. In the theoretical studies, the majority of the limitations are highly dependent on the computational power available, and some of the most advanced ab initio and modern first-principles-based approaches could combine computational efficiency with model accuracy for investigating molecular properties and ion-hydration effects.

Traditionally, the improvement of model potentials is sought by adding physical terms that take into account the many-body influence, normally neglected since the two-body terms comprise most of the interaction energy [19,21,24,25,27,28,30,35,47,48,49,50,51,52,53,54]. This makes the potential much more flexible, presenting less error in conflict zones, although when more parameters are taken into account, part of the meaning assigned to each term is lost and the potential increasingly resembles a black box that makes it more difficult to interpret. More recently, the revolution of machine-learning techniques in the field makes such tools necessary components in improving decisions to be made during the model building process, such as choices of input training set configurations, level of electronic structure theory, etc., that influence the quality of results, and hence should be carefully considered [33,55,56,57,58,59]. The associated design choices typically require the expertise and intuition of experienced scientists; nevertheless, machine-learning approaches could automate and accelerate analysis in a purely data-driven fashion [60,61,62]. Of course, the level of model accuracy/precision is always dependent on the type of application or interest you have, and is traditionally restricted by the limited computational power available.

A reliable potential model must perform well in all ranges from clusters with a few water molecules to nano-droplets. With the intention to find the best approach, investigations are carried out in halide-water systems of increasing size both at the equilibrium points and outside of them, which will tell us which of all methodologies, approaches and models under study is the most reliable for further molecular dynamics simulations. Thus, this work presents a comparison of four polarizable data-driven model potentials (namely, i-TTM2, i-TTM3, i-TTM4, and i-MBpol) together with interaction energies obtained from electronic structure calculations using different type/rung density functionals, such as TPSS, M06L, M062X, B3LYP, LC-ωPBE, CAMB3LYP, and ω-B97XD, as implemented in the Gaussian package [63], considering dispersion corrections [64,65,66] and results from reference wave-function-based calculations at DF-MP2 level of theory [67,68]. All these computations were performed for $F^{-}$, ${\mathrm{Cl}}^{-}$, ${\mathrm{Br}}^{-}$, and $I^{-}$ halide ions in selected size water clusters, containing up to 54 water molecules.

The present work has the following distribution: In the first section, we present a brief description of the computational details of the methods and models used in the study. In the next section, the results obtained are presented and discussed starting with the reference data obtained from DF-MP2 calculations, the different data-driven interaction models, and the DFT/DFT+D energies. Finally, given the importance of knowing the behavior out of equilibrium, an analysis of the best-performing approaches and models is carried out by scanning the position of the halide ion in the water clusters. Some conclusive remarks are reported in the last section.

## 2. Computational Details, Results and Discussion

### 2.1. Building Up Data-Driven Interaction Models

Through the many-body expansion formalism [69], the total energy of the n-bodies/monomers system, $E_{n}$, can be expressed as a sum over higher n-body (nB) energies, $E^{\left(nB\right)}$, $E_{n}=\sum_{n}E_{i}^{\left(1B\right)}+\sum_{n}E_{ij}^{\left(2B\right)}+\sum_{n}E_{ijk}^{\left(3B\right)}+\cdots +E^{\left(nB\right)}$.

In our case, for a halide anion and *n*-water molecules system, the potential energy form reads

$$V_{n}=\sum_{n}V^{\left(1B\right)}+\sum_{n}V^{\left(2B\right)}+\sum_{n}V^{\left(3B\right)}+\cdots +\sum_{n}V^{\left(nB\right)}=\sum_{n}\left(V_{W}^{intra}+V_{W-W}^{inter}+V_{i-W}^{inter}\right),$$

where $V^{\left(1B\right)}$ is the intramolecular distortions of each water molecule ($V_{W}^{intra}$), the $V^{\left(2B\right)}$ contains all 2B halide anion–water and water–water pairwise intermolecular interactions, while $V^{\left(3B\right)}$, ⋯, $V^{\left(nB\right)}$ are the 3B and higher-order nonadditive interaction terms, respectively.

Here, the $V_{W}^{intra}$ term is represented by the Partridge Schwenke potential [70], while the intermolecular water–water interactions ($V_{W-W}^{inter}$) with any of the Thole Type Model (TTM) such as potentials (TTM2, TTM3, TTM4, and MB-pol) [48,50,51,71,72]. In turn, the ion–water intermolecular interaction ($V_{i-W}^{inter}$) is expressed as a sum of 2B short- and long-range contributions, and many-body polarization terms as described by the TTM model [71], $V_{i-W}^{inter}=V_{rep}^{2B}+V_{disp}^{2B}+V_{TTM,elec}^{2B}+V_{TTM,ind}^{nB}$. Briefly, the extended TTM model [71] utilizes for the water three geometry-dependent points charges calculated from the Partridge–Schwenke ab initio dipole moment surface (DMS) [70], two positive smeared charges at the hydrogen atoms and a negative smeared charge shifted from the oxygen atom to the M-site (located near the O atom and along the bisector of the HOH bond angle) [71], and also three polarizable sites at each atom with and an increased damping between the intramolecular dipoles on the H-atom, while a negative smeared charge and a polarizable site are placed on the halide anion. The $V_{TTM,elec}^{2B}$ represents the 2B permanent charge–charge electrostatic contributions, while the $V_{TTM,ind}^{nB}$ includes higher-order induced dipoles interactions, which are all described by a classical many-body polarization model [35,71,73]. The $V_{rep}^{2B}$ term is the repulsive energy between pairs of anion and oxygen or hydrogen atoms, expressed as $\sum_{i,j=\left(O/H\right),X^{-}}A_{ij}e^{\beta_{ij}R_{ij}}$, while $V_{disp}^{2B}$ describes the dispersion energy as a sum of pairwise interactions between ${\mathrm{OX}}^{-}$ and ${\mathrm{HX}}^{-}$, $\sum_{i,j=\left(O/H\right),X^{-}}\frac{C_{6}^{\left(ij\right)}f\left(R_{ij},\delta_{ij}\right)}{R_{ij}^{6}}$, with f(R,δ) being the Tang–Toennies damping functions [74], and $C_{6}^{OX^{-}}$ and $C_{6}^{HX^{-}}$ are the dispersion coefficients. The $\delta_{ij}$ parameters determine the shape to the f(R,δ) damping functions, and were set equal to the $\beta_{ij}$ of the corresponding Born–Mayer functions [75].

**Figure 1:**
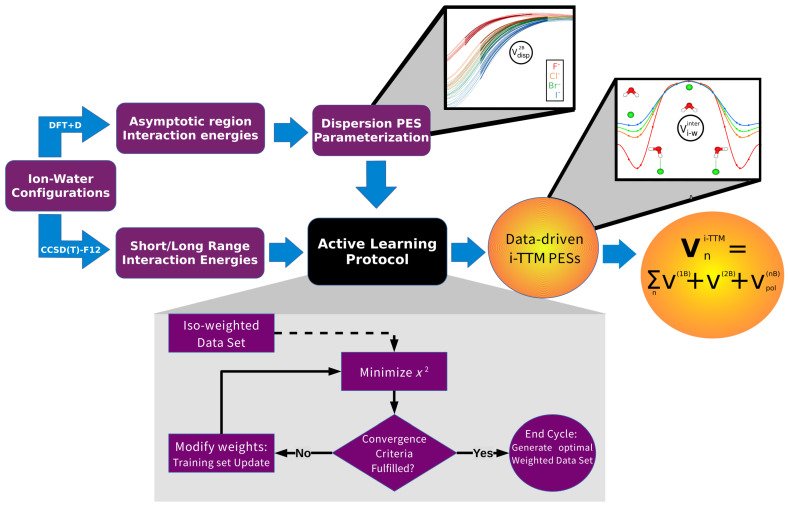
Full automated flowchart of the PES’s parameterization process.

All $C_{6}$, *A*, and β parameters have been determined by their fit to energies from DFT+D and CCSD(T)-F12 level of theories. In particular, the parameterization procedure, shown in Figure 1, consists of a two-stage process. The $C_{6}$ coefficients impose the correct asymptotic behavior, and were evaluated first by the fit to energy data obtained from LC-ωPBE/AVTZ calculations [63], employing the exchange-hole dipole moment (XDM) model [64,76]. For the ion TTM contributions to the interaction, the atomic dipole polarizability of each halide ion was obtained from CCSD(T)/t-aug-cc-pV5Z/t-aug-cc-pV5Z-PP calculations [35], while those for the O and H atoms were obtained from ref. [71]. The remaining model’s parameters were adjusted to the interaction energies at CCSD(T)-F12b/VQZ-F12(-PP) level of theory calculations, including basis set superposition error (BSSE) corrections via the counterpoise (CP) method [77]. For each $X^{-}$$H_{2}$O cluster, interaction energies were computed in a grid of near 500 points distributed equidistantly along the *R* (anion–water (oxygen) distance), θ (polar angle), and ϕ (azimuthal angle) coordinates.

Specifically, during the fitting procedure the linear and nonlinear parameters were optimized using linear regression through singular value decomposition, and the simplex algorithm, respectively. The weighted sum of the squared residuals, given as

$$\chi^{2}=\sum_{N\in T}w_{N}[{\left(V_{i-W}^{\mathrm{inter}}\left(N\right)-\Delta E^{\mathrm{ref}}\left(N\right)\right]}^{2},$$

were computed and minimized, with $\Delta E^{\mathrm{ref}}$ being the reference energies, and N the number of configurations in the training set T. The weight-factors, such as $w_{N}\left(E\right)={\left(\frac{1}{E-E_{min}+DE}\right)}^{2}$, where $E_{min}$ and DE correspond to the minimum energy in the T, and the range of favorably weighted energies, respectively, are traditionally introduced to overcome difficulties in the fit when some space regions need a larger number of points to have a good representation. Usually, this can be also solved by adding new points in such regions of interest; however, this repetitive (forward–backward) procedure turns out to be a time-consuming and computationally expensive task.

Nowadays, machine-learning schemes and data processing tools offer the possibility to also automate the most repetitive tasks in conventional procedures, accelerating or even improving the prediction of parameters [61,62]. Thus, in this work, we explore the application of a fully automated approach for the generation of weighted datasets based on the concept of active learning. Our active learning scheme improves the results obtained from an arbitrary pool of initial data without the calculation of new points, allowing us to weight in an efficient and smart way the potential configurations, reducing the training set size, and thus minimizing the computational cost. The flowchart is displayed in the inset plot of Figure 1, which consists of a pool of equidistant configurations points in R,θ, and ϕ coordinates of each $X^{-}$$H_{2}$O dimer, summarized below:

- We start from a pool of fixed points, in our case those points are equidistant in all R,θ, and ϕ coordinates.
- For each iteration, a minimization of the parameters for the target function $\chi^{2}$ is performed, using a simplex procedure.
- Once the minimization has ended, we check the fulfillment of the convergence criteria, such as the maximum number of steps, minimum average error, and certain error value, along the whole curve to see if it reached convergence.
- If none of the convergence criteria have been reached, the error at each point is obtained.
- For the *N* points with an error larger than a predefined percentage of the maximum error value, we increase their weights in a gradual descent manner throughout the iterations.
- Minimization is performed with the new training set of weights.
- This procedure is repeated until convergence is achieved.

In this way, we generated four families of data-driven $V_{n}$ PESs (see data given in supplementary material), namely i-TTM2, i-TTM3, i-TTM4, and i-MBpol, depending on which TTM water model we chose, to represent in an efficient manner from on a bottom-up approach the halide–water interactions in larger $X^{-}($$H_{2}$O${)}_{n}$ systems, with X being F, Cl, Br, and I anions.

### 2.2. Evolutionary Programming Procedure and Selected Optimal Reference Structures

Given the availability of the i-TTM potential models, halide($H_{2}$O${)}_{n}$ clusters containing an increasing number of water molecules are considered, and their structuring is investigated through optimal potential configurations. Looking for systems with an increasing number of water molecules, we decided to pick some concrete numbers corresponding to *n* equal to 1, 2, 4, 8, 16, 20, 24, 36, 48, and 54 water molecules, which allows having a large distribution of water nanoclusters to explore trends or patterns in the growth of the clusters during the anion hydration/solvation process.

Searching for energy minima in high-dimensional potential surfaces is a challenging task. As the number of atoms in the system increases, its degrees of freedom increase too, which leads to high computational cost if we want to explore the whole configurational space. To deal with this, an evolutionary algorithm (EA) [78,79,80,81] is used in this work. The genetic algorithm is based on natural selection, and begins with an initial guess, and applies modifications, creating a population of possible solutions; these solutions “compete” against a fitness function that we want to optimize, and we retain the best candidates that are the parents for the next generation. In the second step, we create a new population by mixing the values of the previous best solutions (reproduction) and giving some random values (mutation), and then we repeat the previous step, keeping the best individuals of that generation. This process continues until we reach the optimization of the function. For structure optimization, the fitness function is the potential energy surface we want to explore, and the variables we mix in every generation are the coordinates of the atoms of the system.

Such algorithms present an efficient way to solve numerical optimization problems in multi-dimensional space, as gradients of the potential are not needed, while the search space-narrowing is automatically realized by the self-adaptation of mutation [78,79,80]. Briefly, we start with an initial generation of M individuals (for each $X^{-}{\left(H_{2}O\right)}_{n}$ cluster, with X = F, Cl, Br, and I in our case). Each individual is characterized by a pair of real vectors ($\chi_{i},\eta_{i}$${)}_{i=1-M}$, containing the Cartesian coordinates, $\chi_{i}$, of all cluster atoms, and the strategy parameters, $\eta_{i}$ for all individuals, which controls the evolution of the dispersion of the population in time. The initial coordinates $\chi_{i}$ are chosen randomly in the interval (0, Δ), with $\eta_{i}$ = 1 and Δ being a displacement factor that increases the revolution power. Each parent set ($\chi_{i},\eta_{i}$) evolves to generate a new population according to the evolution law: $\chi_{i}^{\prime }\left(j\right)=\chi_{i}\left(j\right)+\eta_{i}\left(j\right)N_{j}\left(0,1\right)$ and $\eta_{i}^{\prime }\left(j\right)=\eta_{i}\left(j\right)\exp\left(\tau^{\prime }N\left(0,1\right)+\tau^{\prime \prime }N_{j}\left(0,1\right)\right)$, where N(0,1) and $N_{j}\left(0,1\right)$ are Gaussian random numbers of average zero and standard deviation 1 generated for each *j* coordinate, while $\tau^{\prime }/\tau^{\prime \prime }$ are adjustable parameters. For each individual of the joint parent–child group (2M individuals) *q* (tournament size), opponents are randomly chosen from the 2M−1 individuals, to compare each other, and the individual in each encounter with the lower potential energy wins. The best individuals then become parents for the next generation and so on. Convergence is achieved when the potential energy difference between two consecutive generations is below a threshold value.

As a starting point, we used the minimum energy structures found in the Cambridge energy landscape database [82] for pure water clusters, and generated initial anion–water configurations by replacing one water molecule in all positions with the halide ion using the DENEB software package [83]. We used generations with 100 individuals and a threshold for the energy of 0.01 kcal/mol. The displacement of the atoms’ coordinates is governed by a Gaussian function and is multiplied by a factor of $\delta_{0}$ = 0.15 that controls the size of the movement and $\delta_{j}$ = 0.85 that updates the value in every generation, which means that every following generation moves 85% less than the previous one.

**Figure 2:**
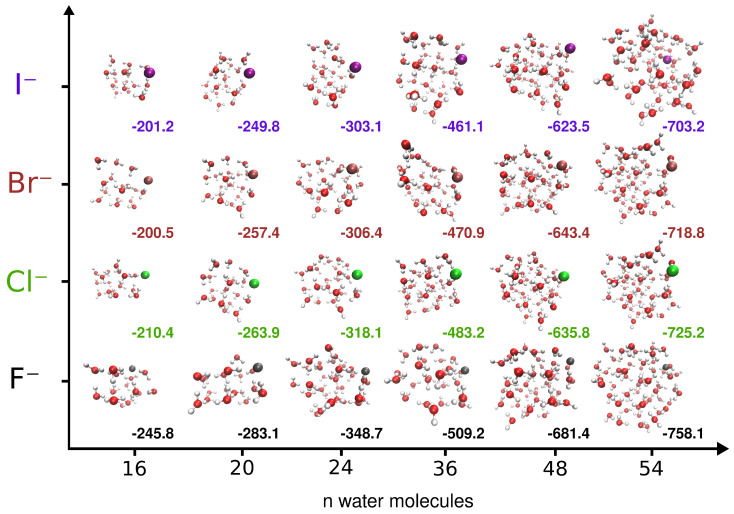
Optimal configurations and energies (in kcal/mol) for the indicated clusters formed by a halide ion and 16, 20, 24, 36, 48, and 54 water molecules.

**Figure 3:**
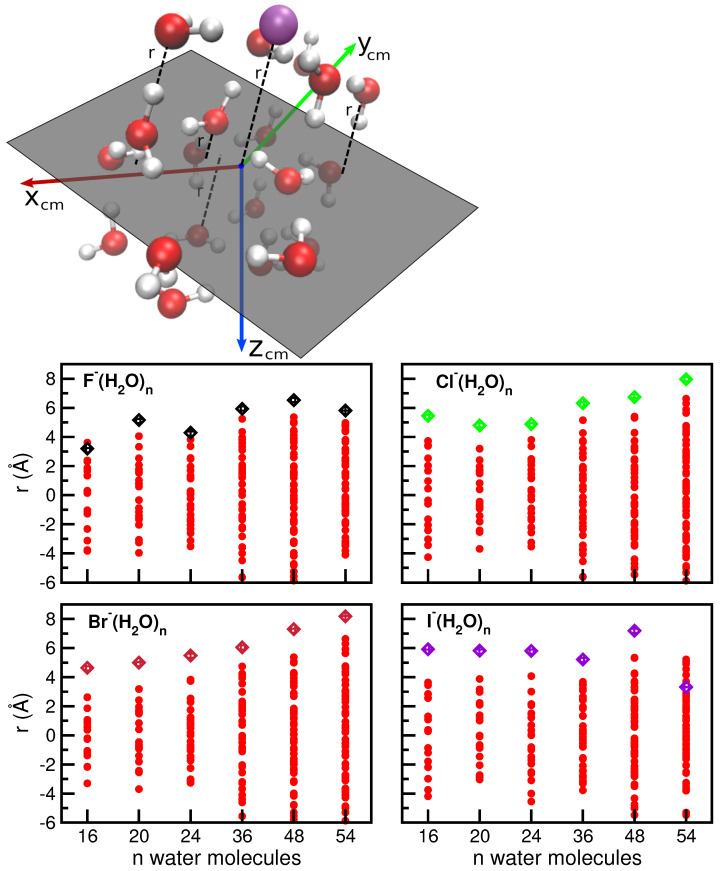
Distances, *r*, of all oxygen (water) atoms (red circles) and halide ion (color diamonds) to the indicated plane (see text) for each *n* water molecules clusters.

In Figure 2, the optimal structures obtained from the EA using the i-TTM4 interaction potentials for all $X^{-}{\left(H_{2}O\right)}_{n=16,20,24,36,48,54}$ clusters under study are shown, together with their corresponding energies, while their geometry coordinates are given in the supplementary material. For the small halide–water systems of up to eight water molecules, the preference of anions to be on the water cluster surface has been proven by previous studies [35,41,84,85]. As can be seen in Figure 2, for all cases the ion presents a tendency to stay at the surface of the cluster. In Figure 3, we defined a plane perpendicular to the line defined by the center of mass of the cluster and the ion position, containing the center of mass point (see upper panel), and we displayed (lower panels) the distances, *r*, from this plane of each oxygen atom of the water molecules or halide ion of the indicated cluster. One can see that the *r* values for the ions are larger than those for oxygens, with the only exception to this rule being found in the $I^{-}($$H_{2}$O${)}_{54}$ cluster, where although it is found near the limits of the cluster, already allows it to be partially surrounded by a few water molecules. However, as the number of water molecules in the cluster increases, the identification of one global minimum becomes less significant, as multiple structures will likely be important for a physical description of the system.

### 2.3. Electronic Structure Calculations and Reference Energy Data

Looking for a computationally accessible wave-function based method to obtain the reference energies for large halide–water clusters with up to 54 water molecules, we chose the density fitting DF-MP2 computations, as implemented in the MOLPRO package [68,86]. Following our previous studies on small-size $X^{-}{\left(H_{2}O\right)}_{n=1-8}$ clusters [35,85], where high-level theories, such as CCSD(T) or explicitly correlated CCSD(T)-F12 calculations can be carried out, we found that DF-MP2 performs reasonable well, and could be a computationally efficient method for larger systems under study. Thus, for the F, Cl, O, and H atoms, we employed all electron aug-cc-pVTZ (AVTZ) basis sets [68,87,88], while for Br and I we used the ECP10MDF and ECP28MDF small-core effective core potentials in conjunction with the aug-cc-pVTZ-PP(AVTZ-PP) valence basis sets [68,89,90,91], and the standard mp2fit auxiliary basis sets for density fitting in the DF-MP2 calculations [67,68]. The interaction energies for each system were calculated as the difference between the total energies of the $X^{-}$($H_{2}$O${)}_{n}$ cluster and the sum of the energies of the water cluster and $X^{-}$ ion, $\Delta E=E_{X^{-}{\left(H_{2}O\right)}_{n=16-54}}-E_{{\left(H_{2}O\right)}_{n=16-54}}-E_{X^{-}}$.

In order to assess comparable alternative first-principles methodologies, we also carried out DFT-based calculations using the Gaussian16 package [63] with the additional dispersion corrections computed by the DFT-D3 and DFT-D4 programs [92,93]. We chose to employ different density functionals from various groups/rungs (according to the Jacob’s ladder classification), such as TPSS [94] and M06L [95] (meta-GGAs/3rd rung), B3LYP [96], and M06-2X [97] (hybrid/meta-GGAs/4th rung), and CAMB3LYP [98], LC-ωPBE [99] and ωB97XD [100] (range-separated hybrid). All DFT computations were carried out with the AVQZ basis set and for the numerical integration, the ultrafine grid was used. In turn, the DFT approaches were corrected, including different semi-classical dispersion schemes, such as the DFT-D3 and its variants regarding the damping functions available in the literature, the original zero-damping function D3(0) [101] and its modified D3M(0) version [102], the most popular Becke–Johnson damping function D3BJ [65] and a modified version D3M(BJ) [102] with an emphasis on non-equilibrium particularly compressed geometries, as well as the most recently developed DFT-D4 [66] model, including many-body dispersion interactions beyond the pairwise terms. In all electronic structure computations (see data given in the supplementary material) and data analyses, the DENEB toolkit utilities [83] were employed.

### 2.4. Comparative Analysis of the Different Halide–Water Potentials: Equilibrium Structures

**Figure 4:**
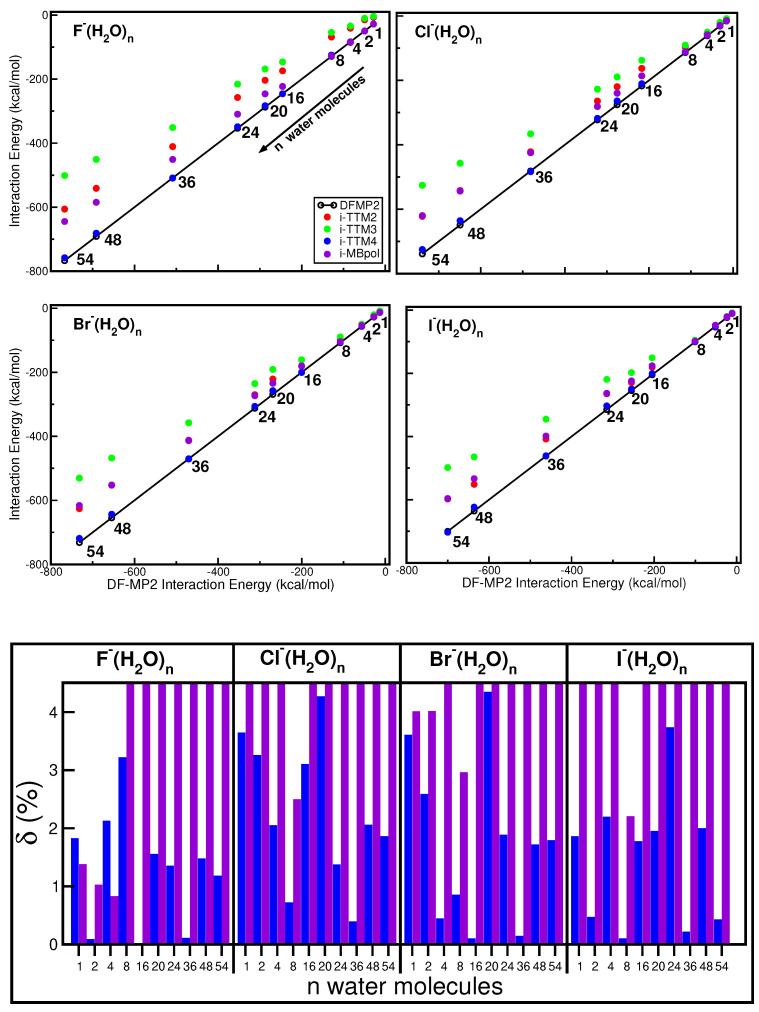
Correlation plots (upper and middle panels) and percentage (%) difference between the ion–water potential (i-TTM2, i-TTM3, i-TTM4, i-MBpol) values and the DF-MP2 energies at the corresponding optimal geometry structure of each indicated $X^{-}{\left(H_{2}O\right)}_{n}$ cluster.

Correlation plots with interaction energies from the DF-MP2 calculations and potential values from the i-TTM2, i-TTM3, i-TTM4, and i-MBpol halide–water models are presented in Figure 4. Energies for the different $F^{-}$ to $I^{-}$ ions clusters, with *n* = 1, 2, 4, 8, 16, 20, 24, 36, 48, and 54 water molecules, were obtained at their optimal energy structures (see Figure 2).

One can see that all model potentials show similar behaviors, with i-TTM3 having larger deviations than the i-TTM2, i-MBpol, and i-TTM4 ones. We should note that i-TTM2 energies are very close to the i-MBpol for all ${\mathrm{Cl}}^{-}{\left(H_{2}O\right)}_{n}$, ${\mathrm{Br}}^{-}{\left(H_{2}O\right)}_{n}$, and $I^{-}{\left(H_{2}O\right)}_{n}$ clusters studied, while this is not the case for the $F^{-}{\left(H_{2}O\right)}_{n}$ ones. In all type and size cluster cases, the i-TTM4 provides energy values that are in closer agreement with the DF-MP2 energies. Energy differences (δ in %) between the i-TTM4 and i-MBpol potential values of each $X^{-}{\left(H_{2}O\right)}_{n}$ cluster structure from the corresponding DF-MP2 reference energies are shown in the lower panel of Figure 4. The calculated errors are found to be less than 3.2, 4.3, 3.6, and 3.7% for the $F^{-}{\left(H_{2}O\right)}_{n}$, ${\mathrm{Cl}}^{-}{\left(H_{2}O\right)}_{n}$, ${\mathrm{Br}}^{-}{\left(H_{2}O\right)}_{n}$, and $I^{-}{\left(H_{2}O\right)}_{n}$ i-TTM4 potentials, respectively, while somehow larger deviations, up to 16%, were obtained for the i-MBpol values. The main difference between the i-TTM4 and i-MBpol model potentials is that the latter incorporates the MB-pol water–water model [72], which includes explicitly short-range three-body terms, while the TTM4-F water model [71] contains only pair (two-body) short-range components, with the higher-body contributions described in both models by induction terms.

### 2.5. Comparative Analysis of DFT/DFT+D Approaches: Equilibrium Structures

**Figure 5:**
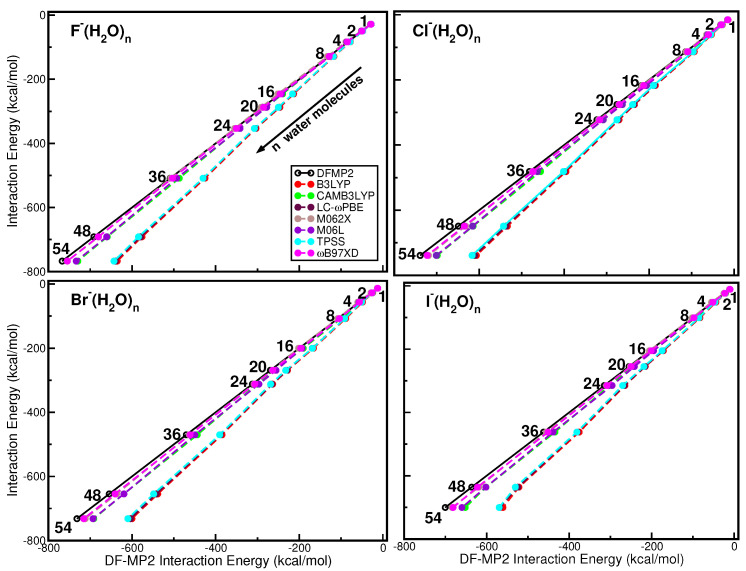
Correlation plots for the indicated DFT values and the DF-MP2 interaction energies at the corresponding optimal geometry structure of each indicated $X^{-}{\left(H_{2}O\right)}_{n}=1-54$ cluster.

In the search for a computationally affordable method for studies in which the speed of calculation is important, in addition to the model potentials, the DFTs could present an alternative, which is fast enough to be used in direct (on-the-fly) computational simulations. Dozens of DFT functionals exist in the literature along with a variety of dispersion corrections and can be classified according to the approximations they use. On the basis of previous studies on smaller $X^{-}{\left(H_{2}O\right)}_{n}$ systems [35,85], in this work we decided to consider two meta-GGA functionals, TPSS and M06L; one hybrid-GGA, B3LYP; one hybrid-meta-GGA, M062X; and three range separated hybrids, CAMB3LYP, LC-ωPBE and ωB97XD. Figure 5 shows the correlation plots of the DF-MP2 and DFT interaction energies of these functionals. One can see that all functionals show the same behavior for all $X^{-}{\left(H_{2}O\right)}_{n}$ systems. Among the functionals with the “best-performance” for the four ion–water clusters is ωB97XD, which intrinsically includes the D2 dispersion [100], followed by the M062X, M06L and CAMB3LYP, while TPSS, LC-ωPBE and B3LYP show significant differences.

Dispersion plays an important role in these systems, since long-distance interactions are important both in hydrogen bonds between water molecules and in interactions between water and ions, especially in those ions whose electron cloud is very large, e.g., ${\mathrm{Br}}^{-}$ and $I^{-}$. Therefore, we carried out calculations for all remaining functionals by adding different semi-classical dispersion corrections, such as D3 with its variants D3(0) and D3(BJ) [65,101], as well as the modified versions D3M(0) and D3M(BJ) [102], and the recent D4 schemes (Damped Axilrod-Teller-Muto D4(ATM) and many-body dispersion D4(MBD)) [66].

In the left-side panels of Figure S1, we present the percentage (%) error, δ, of the indicated selected best-performing DFT/DFT+D energies and the i-TTM4 model values from the corresponding DF-MP2 energies for each halide–water cluster of up to 54 water molecules, while in the right-side panels of the figure a graphical representation of the errors (in %) of the four best-performing DFT/DFT+D functionals or the i-TTM4 model from the DF-MP2 energies for each cluster with *n* = 16–54 are displayed.

The importance of adding dispersion corrections in the DFT approaches for these systems can be seen. We found that the error increases as the number of water molecules increases, indicating the inability of the DFT functionals to deal with additive long-range interactions, while by adding dispersion such errors have been balanced or even eliminated. In general, we obtain better results when adding the corresponding corrections, except in the case of CAM-B3LYP, for which dispersion worsens their energies, as well as the M062X+D3(0) for the smaller size clusters. In particular, among the TPSS approaches, D3 and D4 show better results for $F^{-}$/${\mathrm{Cl}}^{-}$/${\mathrm{Br}}^{-}$ and $I^{-}$ water clusters, respectively, while the M06L dispersion corrections yield in general rather high deviations. Next, dispersion schemes seem to work well for the B3LYP, with the D4 notably improving results, showing a balanced behavior for all sizes and halide–water systems studied, with errors of less than 1%, with LC-ωPBE+D3M following with errors of around 1%, and then TPSS+D3(D4) with slightly higher than 1%.

### 2.6. Overall Comparative Analysis: Non-Equilibrium Configurations

**Figure 6:**
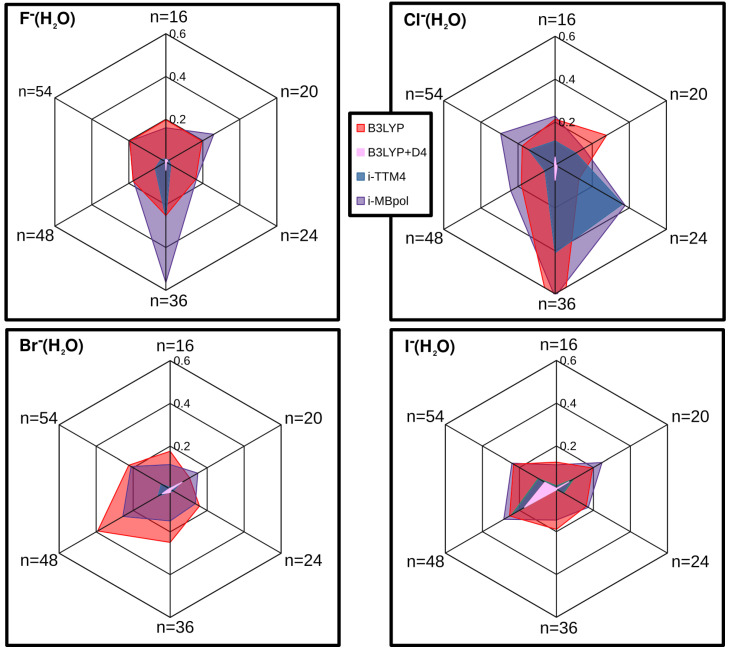
Average errors (normalized) for the indicated DFT+D approaches and model potentials obtained from the scans of the different halide–water clusters (see Figures S2–S5 in SI).

In Figure S1, one should also notice the performance of the i-TTM4, especially for the $F^{-}$($H_{2}$O)${}_{n}$ clusters. However, we should mention that the good behavior of the i-TTM4 potential compared to the other potentials could be due to the fact that the minimum energy geometries have been optimized using that potential. Thus, to further test the DFT+D and model potentials, a study was carried out by moving the ions out of their lowest energy configuration. We started by placing the ion into the center of the mass of the cluster structure, $R_{ion-water}$ = 0, and moved it with variable size steps along the direction defined by the line joins it to the ion’s position at the corresponding minimum energy configuration. In Figures S2–S5 (see Supplementary Materials), we display such scans for all F, Cl, Br, and I halide anions and 16, 20, 24, 36, 48, and 54 water molecules clusters. In addition to the four i-TTM potentials, the energies are presented at the center of mass and at intermediate points for DF-MP2, B3LYP and B3LYP+D4(MBD); as such, DFT+D approaches were found to yield the best and balanced performance in all halide ion–water clusters. One can see that the chosen non-equilibrium structures sample various repulsive, attractive and dissociative areas of the potential surfaces, and for all those sizes of halide ion–water clusters, configurations the i-TTM4 and B3LYP+D4 were found closer to the reference DF-MP2 energies. In Figure 6, we summarize all the results obtained for each halide ion for the indicated size water clusters. By analyzing averaged errors along the scans, as in the case of equilibrium structures, we found that the B3LYP+D4 DFT approach yields the best results with errors of less than 0.1 kcal/mol in most cases, indicating clearly the improvement of including dispersion correction in the B3LYP functional. Next, the i-TTM4 potential showed errors of 0.2, 0.4, 0.05, 0.2 kcal/mol for $F^{-}{\left(H_{2}O\right)}_{n}$, ${\mathrm{Cl}}^{-}{\left(H_{2}O\right)}_{n}$, ${\mathrm{Br}}^{-}{\left(H_{2}O\right)}_{n}$ and $I^{-}{\left(H_{2}O\right)}_{n}$ clusters, respectively.

## 3. Conclusions

We have adopted a general protocol scheme to access promising model potentials and DFT/DFT+D approaches against wave-function-based reference data. First of all, we built up data-driven bottom-up interaction potentials for various halide ion–water systems, such as $F^{-}$, ${\mathrm{Cl}}^{-}$, ${\mathrm{Br}}^{-}$, and $I^{-}$. We explored the application of a fully automated approach for the generation of optimally weighted datasets, accelerating the prediction of adjusted parameters without adding new energies to the dataset. In general, during the fitting processes, difficulties are commonly present due to the poor grid point representation of the potential surface at some space regions. Our active learning scheme allows us to weight in an efficient and smart way the potential configurations, increasing their values in those areas, where the prediction presents higher errors, reducing the training set size, and minimizing the computational cost.

In this way, we presented four i-TTM families of parameterized model PESs trained on $X^{-}$$H_{2}$O CCSD(T)-F12 data, including implicitly many-body polarization contributions terms. Our intention is to employ such model interaction potentials in computationally high-demanding dynamics simulations for reliable nanoclusters’ and bulk property investigations. Thus, we have reported new benchmark datasets by increasing the size of the clusters, up to 54 water molecules. We span a broad range of different representative configurations for each benchmark structure, from equilibrium, moderate binding to nonbinding ones to ensure an overall description of the interactions at different levels of theory.

By analyzing the energy differences between electronic structure wave-function-based methodologies, such as DF-MP2, and various DFT+D approaches, we evaluated the performance of the DFT functionals in the presence/absence of dispersion corrections, as well as the new i-TTM model potentials. We highlighted the importance of adding dispersion to the DFT approaches, providing improved data. We found that the B3LYP+D4 functional and the ab initio-based i-TTM4 model yields the best overall results in comparison to the DF-MP2 reference data for all clusters sizes and structures studied. Such an outcome reveals that less demanding computational methods could present alternatives to adequately describe halide–water interactions in finite-size clusters. As such systems are large enough, we should underline the importance of considering well-converged reference energy data, as well as a variety of representative configurations for cross-checking computational approaches. Investigations in this direction could further benefit from the application of active learning techniques to generate the automatic sampling of more objective representative datasets.

Moreover, finite-size clusters can serve as model microsolutions, so the reported stable potential structures for each cluster, through evolutionary programming algorithm energy optimizations, should be expected to influence halide ions’ hydration. All their structures show an on-surface preference of the halide ion as clusters grow, indicating a competition between the ion–water and water–water interactions. However, such a classical description of energetics and structuring of halide–water clusters should be further extended to include nuclear quantum and thermal effects. Such additional information could be useful for investigating the transition from molecular nanoscale aggregates to dissolved anions. Currently, path-integral molecular dynamics (PIMD) simulations are in progress for these halide–water clusters, employing the current data-driven i-TTM4 model potentials to explore the anions’ distribution in water clusters of increasing size.

## Data Availability

The data supporting reported results are available from the corresponding author upon request. The parameters of potential models, electronic structure DFMP2 and DFT/DFT-D calculations and i-TTM models energies, as well as optimized geometry coordinates, can be see in the Supplementary Materials.

## Supplementary Materials

The following supporting information can be downloaded online. Figure S1: Errors δ (in %) for the indicated DFT/DFT+D functionals and i-TTM4 model. Figure S2: Scan of fluorine ion with clusters of 16, 20, 24, 36, 48, and 54 water molecules. Figure S3: Scan of chlorine ion with clusters of 16, 20, 24, 36, 48, and 54 water molecules. Figure S4: Scan of bromine ion with clusters of 16, 20, 24, 36, 48, and 54 water molecules. Figure S5: Scan of iodine ion with clusters of 16, 20, 24, 36, 48, and 54 water molecules.
