# Supplementary material for: A Benchmark Protocol for DFT Approaches and Data-Driven Models for Halide-Water Clusters
Source: Molecules. 2022 Mar 2;27(5):1654. doi: 10.3390/molecules27051654 (PMC8924895; doi:10.3390/molecules27051654)
Supplement: Supplementary file 1 [file molecules-27-01654-s001.zip › SI-Figs-S1-S5.pdf]

Article

A Benchmark Protocol for DFT approaches and Data-Driven Models for Halide-Water Clusters.

Raúl Rodríguez-Segundo [1,2,§], Daniel J. Arismendi-Arrieta[3] & Rita Prosmiti [1,*]

[1] Institute of Fundamental Physics (IFF-CSIC), CSIC, Serrano 123, 28006 Madrid, Spain
[2] Atelgraphics S.L., Mota de Cuervo 42, 28043 Madrid, Spain
[3] Department of Chemistry, Ångström Laboratory, Uppsala University, Box 538, 75121 Uppsala, Sweden
* Correspondence: rita@iff.csic.es; Tel.: +34-91-5616800 ext 442292
§ Doctoral Programme in Theoretical Chemistry and Computational Modelling, Doctoral School, Universidad Autónoma de Madrid, Spain

Supplementary material

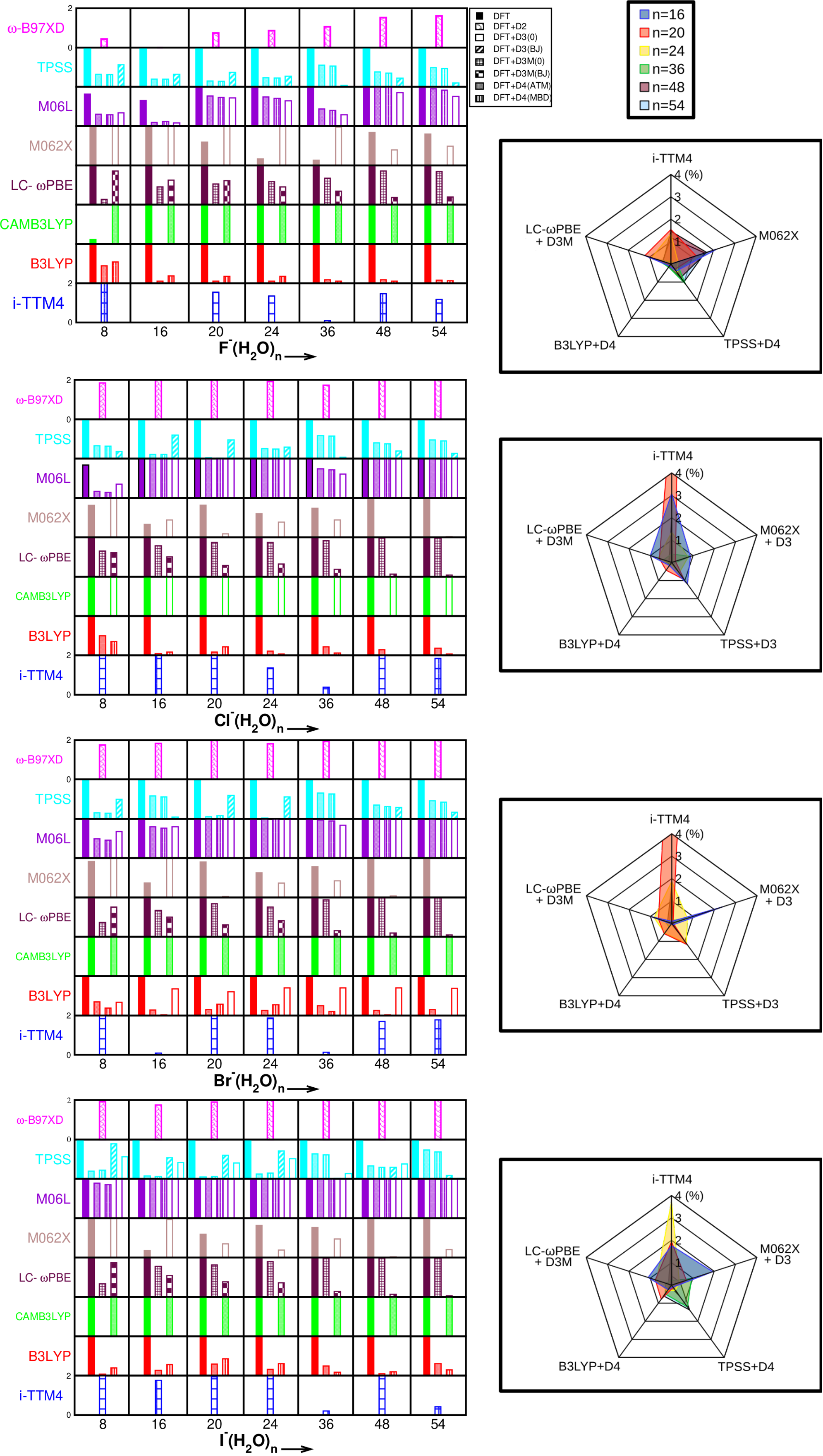

Figure S1. Errors δ (in %) for the indicated DFT/DFT+D functionals and i-TTM4 model.

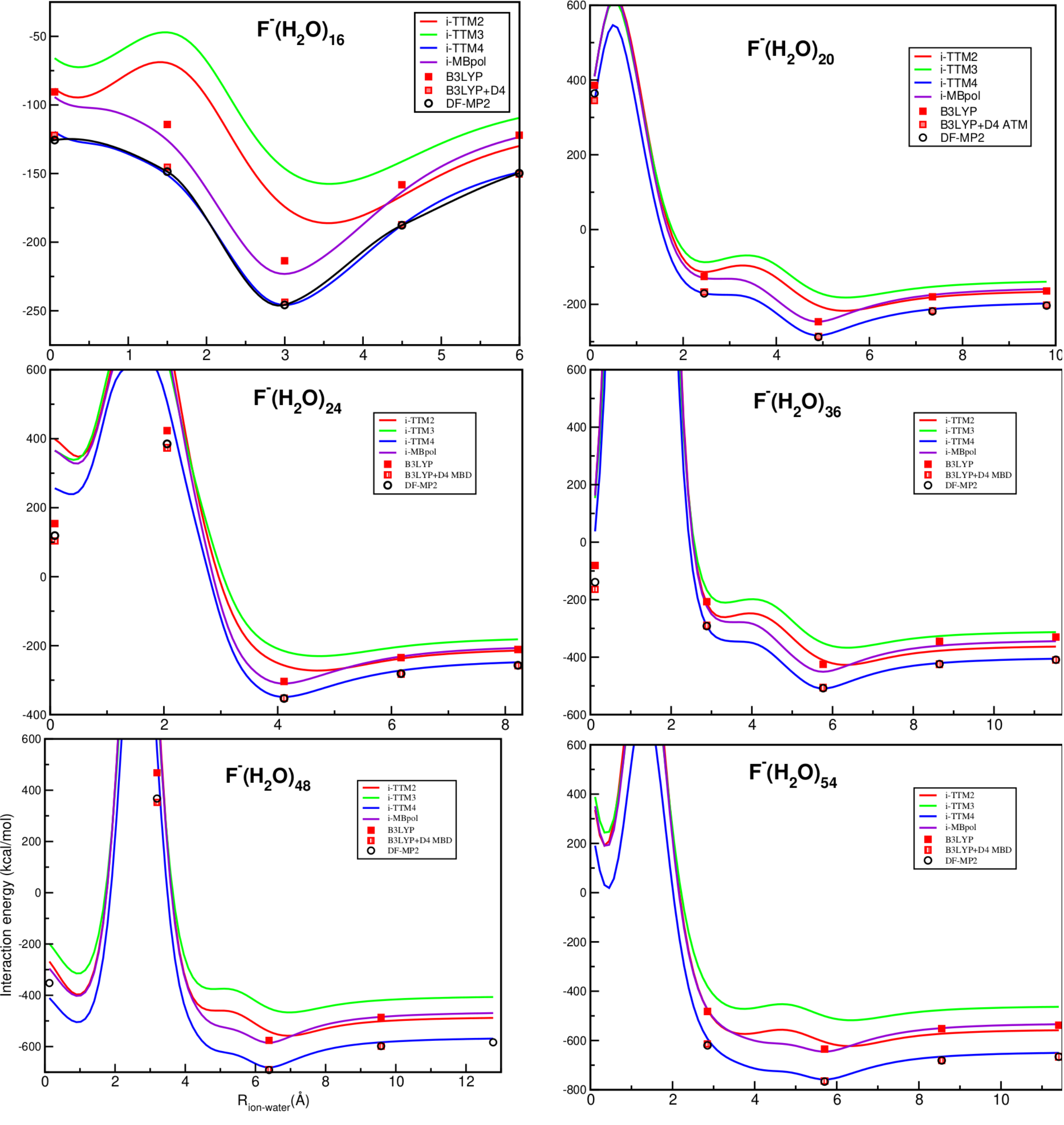

Figure S2. Scan of fluorine ion with clusters of 16, 20, 24, 36, 48, and 54 water molecules

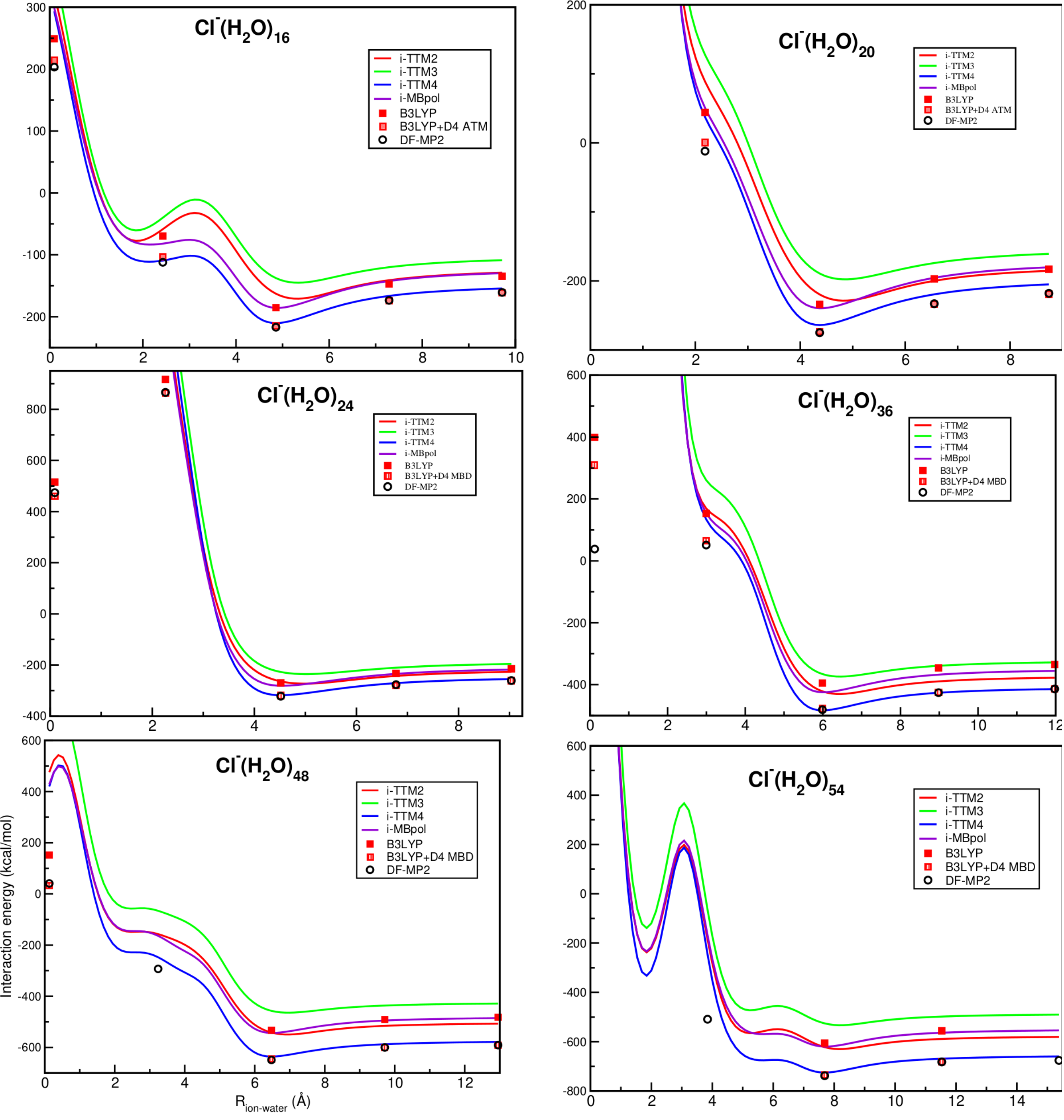

Figure S3. Scan of chlorine ion with clusters of 16, 20, 24, 36, 48, and 54 water molecules

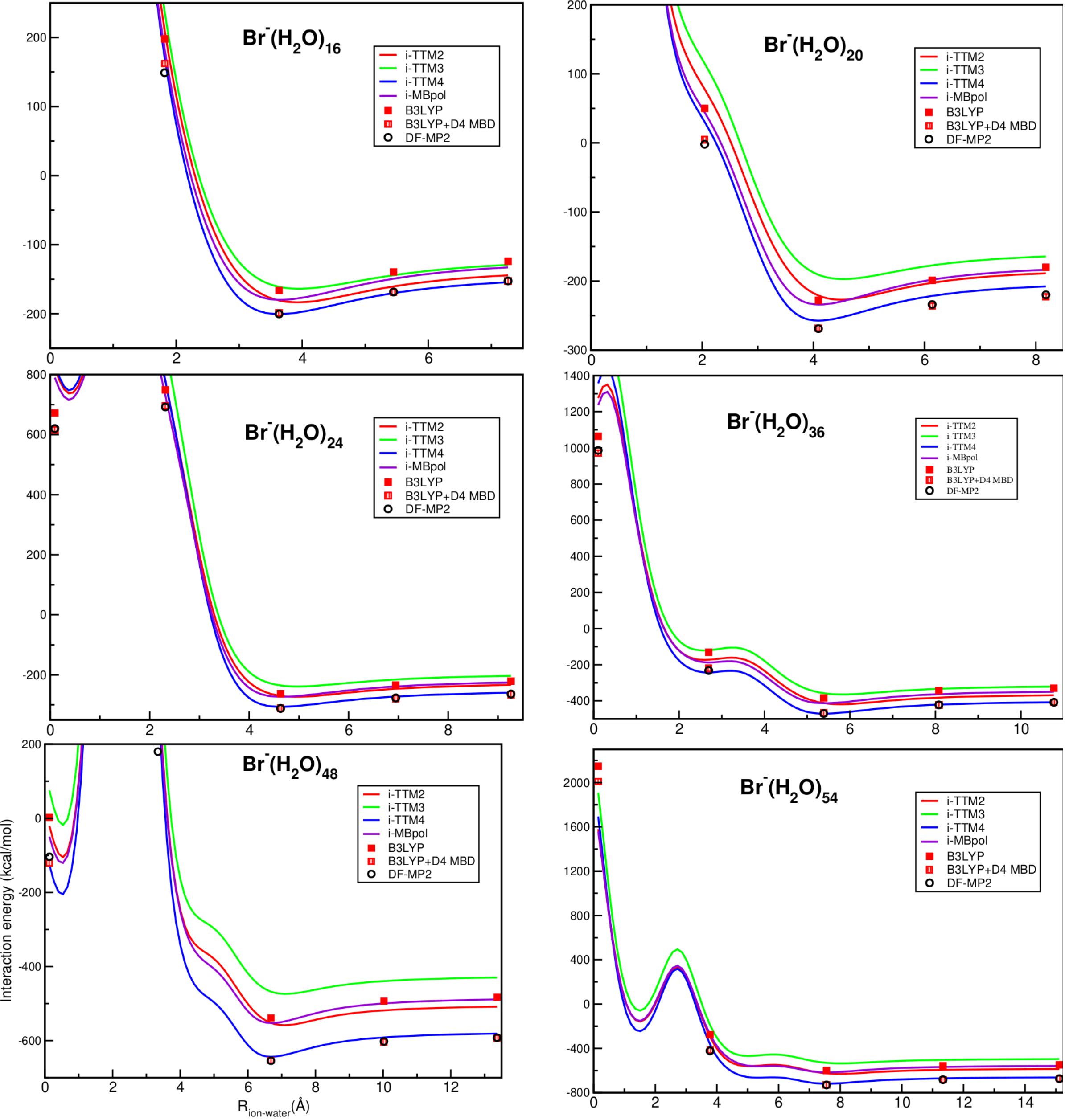

Figure S4. Scan of bromine ion with clusters of 16, 20, 24, 36, 48, and 54 water molecules

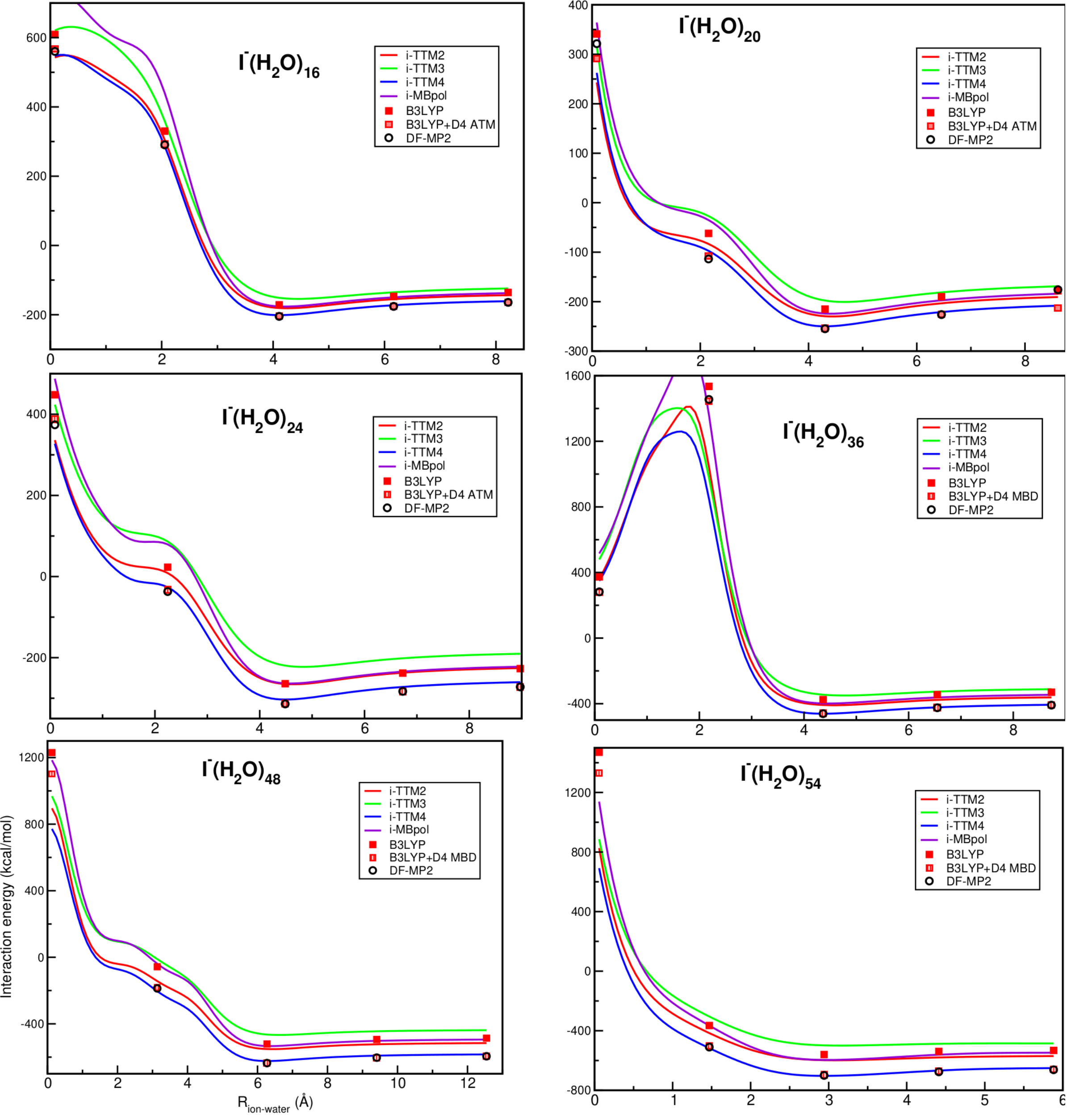

Figure S5. Scan of iodine ion with clusters of 16, 20, 24, 36, 48, and 54 water molecules
